# Type I collagen inhibits differentiation and promotes a stem cell-like phenotype in human colorectal carcinoma cells

**DOI:** 10.1038/sj.bjc.6605143

**Published:** 2009-06-30

**Authors:** S C Kirkland

**Affiliations:** 1 Department of Histopathology, Imperial College London, DuCane Road, London W12 ONN, UK

**Keywords:** colorectal cancer, cancer stem cell, integrin, epithelial–mesenchymal transition, collagen, CD49b

## Abstract

**Background:**

Human colorectal cancer is caused by mutations and is thought to be maintained by a population of cancer stem cells. Further phenotypic changes occurring at the invasive edge suggest that colon cancer cells are also regulated by their microenvironment. Type I collagen, a promoter of the malignant phenotype in pancreatic carcinoma cells, is highly expressed at the invasive front of human colorectal cancer.

**Methods:**

This study investigates the role of type I collagen in specifying the colorectal cancer cell phenotype. The effect of type I collagen on morphology, localisation of cell–cell adhesion proteins, differentiation and stem cell-like characteristics was examined in a panel of human colorectal carcinoma cell lines.

**Results:**

Human colorectal carcinoma cells grown on type I collagen in serum-free medium show an epithelial–mesenchymal-like transition (EMT-like), assuming a more flattened less cohesive morphology. Type I collagen downregulates E-cadherin and *β*-catenin at cell–cell junctions. Furthermore, type I collagen inhibits differentiation, increases clonogenicity and promotes expression of stem cell markers CD133 and Bmi1. Type I collagen effects were partially abrogated by a function-blocking antibody to *α*2 integrin.

**Conclusion:**

Together, these results indicate that type I collagen promotes expression of a stem cell-like phenotype in human colorectal cancer cells likely through *α*2*β*1 integrin.

The human colorectal epithelium is maintained by a population of multipotent adult stem cells that self-renew and execute a multilineage differentiation programme yielding absorptive, mucous and endocrine cells (Brittan and Wright, 2004). Human colorectal carcinomas are heterogeneous and contain differentiated cells (Ho *et al*, 1989), suggesting that cancer stem cells, with a residual capability for multilineage differentiation, propagate colorectal cancer. This concept is supported by the finding that single-cell-derived populations of human colorectal cancer cells have been shown to be both tumourigenic and capable of multilineage differentiation (Kirkland, 1988; Vermeulen *et al*, 2008). Furthermore, several recent studies have isolated a sub-population of cancer stem or tumour-initiating cells from human colorectal cancers, which are functionally characterised by their ability to seed new tumours (Dalerba *et al*, 2007; O'Brien *et al*, 2007; Ricci-Vitiani *et al*, 2007).

Epithelial–mesenchymal transition (EMT) has long been implicated in cancer progression (Thiery and Sleeman, 2006), although EMT-like would more accurately describe the changes seen in most cancers where a full transcriptional reprogramming to a mesenchymal phenotype is rarely observed (Klymkowsky and Savagner, 2009). In human colon cancer, EMT-like changes are observed in cells at the invasive edge, but these appear to be reversible as disseminated cells recapitulate the morphology of the primary tumour (Brabletz *et al*, 2005). Interestingly, EMT-like phenotypic changes have recently been linked with stemness in mammary epithelial cells (Mani *et al*, 2008) and the concept of migratory cancer stem cells has been suggested (Brabletz *et al*, 2005) to describe human colon cancer progression.

The majority of human colon cancers carry mutations that lead to the activation of Wnt signalling, a pathway that also has a pivotal role in normal intestinal stem cell biology (Reya and Clevers, 2005). Despite the underlying genetic background, cells within individual tumours display differential Wnt signalling, suggesting further regulation by the microenvironment. A local loss of basement membrane at the invasive edge has been suggested to expose cancer cells to a different microenvironment, which promotes Wnt signalling (nuclear *β*-catenin expression), EMT-like changes and loss of differentiation (Spaderna *et al*, 2006). Type I collagen is a known component of the microenvironment at the host–tumour interface in human colorectal cancer (Brabletz *et al*, 2004) and is more highly expressed in tumours displaying infiltrative growth compared with those with expansive growth (Oku *et al*, 2008).Type I collagen also reduces cdx-2 expression in human colorectal cancer cell lines *in vitro* (Brabletz *et al*, 2004) and enhances tumourigenicity in human colorectal cancer cells in xenografts (Del-Buono *et al*, 1991). Furthermore, type I collagen promotes a malignant phenotype in pancreatic cancer through *α*2*β*1 integrin (Grzesiak and Bouvet, 2006) and forms part of a 17-gene signature associated with metastasis (Ramaswamy *et al*, 2003).

Recently, the collagen receptor *α*2*β*1 integrin has been shown to regulate stem cell fate in multipotent human colorectal cancer cells (Kirkland and Ying, 2008), suggesting that collagen is involved in the loss of differentiation observed at the invasive front. This study investigates the effect of type I collagen on the phenotype of human colorectal carcinoma cells.

## Materials and methods

### Cells

Three human colorectal cell lines were used in this study: HCA-7 Colony 29 (Marsh *et al*, 1993) termed Colony 29 in this study, Caco-2 (ECACC) and a twice cloned population of HRA-19 cells (Kirkland, 1988), which have been subjected to multiplex PCR analysis (ECACC; Porton Down, Salisbury, UK) to confirm their unique profile. Cell lines were grown in Dulbecco's Eagle's medium (Invitrogen, Paisley, UK) supplemented with 10% foetal bovine serum (FBS) in 7.5% CO_2_.

For experiments, cells were transferred to different serum-free media, which are as follows:

IT: DMEM with 2 mM glutamine and 1 : 100 of ITS-X supplement (Invitrogen)
ITA: IT medium with 10 *μ*g ml^−1^ ascorbic acid
TS: DMEM with 2 mM glutamine, 10 *μ*g ml^−1^ transferrin (Invitrogen) and 5 ng ml^−1^ selenous acid (Sigma, Gillingham, UK).

### Endocrine lineage commitment assay

Lineage commitment experiments were performed as previously described (Kirkland and Henderson, 2001) or with minor modifications.

### Western blotting

Lysates were prepared with non-reducing SDS lysis buffer (New England Biolabs, Hitchin, UK). Equal amounts of protein (RC-DC assay; Bio-Rad, Hemel Hempstead, UK) were separated on 3–8% Tris-acetate gels (Invitrogen) and blotted onto nitrocellulose. Blots were stained with Ponceau S solution (Sigma) to check for equal loading. Blots were blocked with 5% block solution (GE Healthcare, Little Chalfont, UK), rinsed in wash buffer (10 mM Tris-HCl, 0.1 M NaCl, 0.1% Tween 20) and incubated overnight with antibodies in the blot wash. Blots were washed and incubated in horseradish peroxidase (HRP)-linked rabbit anti-mouse antibodies (Dako, Ely, UK) in blot wash for 1 h at room temperature, washed and developed using ECL Plus (GE Healthcare).

### Alkaline phosphatase assay

Subconfluent cells were removed from flasks with trypsin/EDTA solution and added to 10% FBS in DMEM. Cells were washed twice in DMEM and seeded in serum-free medium comprising Dulbecco's Eagle's medium containing transferrin (10 *μ*g ml^−1^), selenous acid (5 ng ml^−1^) and 2 mM glutamine (TS medium). Cells were plated at 0.6 × 10^4^ cells per well (Caco-2), 1.5 × 10^4^ cells per well (Colony 29) or a 1 : 5 split ratio (HRA-19). Cells were seeded into collagen-coated 96-well plates (BioCoat; BD Biosciences, Oxford, UK) or equivalent non-coated plates from BD Biosciences (Biocoat plates are coated with 40–60 *μ*g ml^−1^ rat tail collagen: manufacturer information). Occasional experiments were performed using plates coated overnight at 4 °C with type I collagen (Sigma) (10 *μ*g ml^−1^). After 72 h at 37 °C, alkaline phosphatase activity was measured using *p*-nitrophenyl phosphate (Millipore, Watford, UK); the reaction product *p*-nitrophenol was measured at 405 nm. Cell numbers were determined in replicate wells using WST-1 reagent (Roche, Burgess Hill, UK) as described by the manufacturer. Alkaline phosphatase absorbance values were normalised using these WST-1 values.

### Cell-based enzyme-linked immunosorbent assay

Quantitative analysis of differentiation and stem cell markers was performed using a cell-based enzyme-linked immunosorbent assay (ELISA) on whole cells in 96-well plates broadly as described previously (Versteeg *et al*, 2000). Briefly, equal numbers of cells were seeded onto either collagen-coated 96-well plates (Biocoat; BD Biosciences) or control non-coated plates (BD Biosciences). Cells were seeded as follows: HRA-19 (1 : 5 split ratio) (a single-cell suspension could not be obtained for cell counting): Colony 29 (1.5 × 10^4^ per well) and Caco-2 (0.66 × 10^4^ per well) in the medium as indicated. Cells were grown for the times indicated and then fixed in ethanol for intestinal alkaline phosphatase expression or 2% paraformaldehyde for CD133 and Bmi1 expression. Cells for Bmi1 measurements were further treated with ice-cold methanol for 20 min.

Cells were washed three times with phosphate-buffered saline (PBS) and endogenous peroxidase blocked with 0.3% H_2_O_2_ in PBS for 30 min at room temperature. Cells were then blocked in 10% normal serum (species of secondary antibody), drained without washing and incubated with primary antibody for 3 h at room temperature or overnight at 4 °C (CD133 (Abcam, Cambridge, UK):Bmi1 (New England Biolabs):Intestinal Alkaline Phosphatase (Abcam)). Wells were washed three times with PBS and then incubated with HRP-conjugated secondary antibody for 1 h at room temperature. Finally, wells were washed three times in PBS and once in distilled water and then HRP developed with tetramethylbenzidine (Sigma), reaction stopped with H_2_SO_4_ and absorbance read at 450 nm (620 nm reference). Background levels, in the presence of isotype control antibodies at the same concentration, were deducted from test values. Values were normalised for cell number measured in replicate wells using Cyquant cell proliferation assay (Invitrogen) as indicated by the manufacturer.

To assess the role of *α*2*β*1 integrin in mediating the effects of collagen, some experiments were performed in the presence of MAb Ak7, a function-blocking antibody to *α*2 integrin (Gamble *et al*, 1993).

### Immunofluorescence

Immunofluorescence was performed in eight-chamber slides (Nunc, VWR, Lutterworth, UK). Chambers were coated with type I collagen (50 *μ*g ml^−1^ in 10 mM acetic acid) (Sigma) overnight at 4 °C. Control wells were incubated with 10 mM acetic acid. Solutions were removed and wells dried for 2 h before use. Cells were grown in serum-free medium (TS)-DMEM with 2 mM glutamine, transferrin (10 *μ*g ml^−1^) and selenous acid (5 ng ml^−1^) for 72 h in eight-chamber slides (Nunc).

Cells were fixed in 2% paraformaldehyde followed by methanol to facilitate antibody access to intracellular antigens. Cells were blocked with 10% normal serum (species of secondary antibody), and drained and incubated overnight in primary antibody in 0.1% BSA in PBS (CD133 (Abcam), Bmi1 (New England Biolabs), *β*-catenin (New England Biolabs) and E-cadherin (BD Biosciences)). Secondary antibodies were Alexa^488^-conjugated Rabbit anti-Ms or Goat anti-Rabbit Immunoglobulins (Invitrogen). Cells were washed and counterstained with DAPI (Sigma) before mounting in Permafluor (Thermo Scientific, Loughborough, UK).

### Clonogenicity

Caco-2 cells were removed from flasks with Trypsin/EDTA solution and then added to an equal amount of DMEM with 10% FBS. Cells were washed twice with DMEM, and then suspended in serum-free medium (TS). Cells were filtered through a 40-*μ*m cell strainer (BD Biosciences) to remove clumps and counted. A single-cell suspension of Caco-2 cells was seeded into either BD Biocoat 6 cm dishes or control uncoated dishes in serum-free medium (TS). Cells were fed twice weekly with an 80% medium change for 5 weeks, and then fixed and stained with crystal violet. Colonies >10 cells were counted in the whole of each dish.

## Results

### Type I collagen induces EMT-like changes in human colorectal cancer cell lines

**Figure 1:**
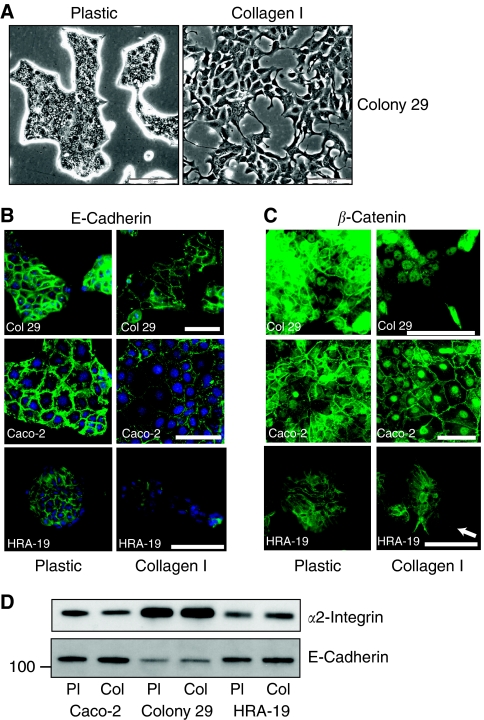
Type I collagen induces EMT-like changes in colorectal cancer cell lines. (**A**) Phase contrast micrographs of Colony 29 cells grown for 48 h in serum-free medium (IT) on plastic tissue culture dishes (plastic) or dishes coated with type I collagen (collagen I). Scale bar=200 *μ*m. (**B**) Immunofluorescent staining for E-cadherin expression in Colony 29, Caco-2 and HRA-19 cells grown for 72 h in serum-free medium (IT) on tissue culture plastic (plastic) or collagen-coated plastic (collagen I). Cells were imaged using fluorescence microscopy. Cells were counterstained with DAPI (blue). Scale bars=100 *μ*m. (**C**) Immunofluorescent staining for *β*-catenin expression in Colony 29, Caco-2 and HRA-19 cells grown for 72 h in serum-free medium (IT) on tissue culture plastic (plastic) or collagen-coated plastic (collagen I). Cells were imaged using fluorescence microscopy. White arrow in HRA-19 collagen panel shows a single cell with nuclear *β*-catenin expression. Scale bars=100 *μ*m. (**D**) Caco-2, Colony 29 and HRA-19 cells were seeded onto tissue culture plastic (Pl) or type I collagen (Col)-coated plastic and incubated at 37 °C. Lysates were prepared after 48 h. Western blot for *α*2 integrin and E-cadherin was performed on 10 *μ*g cell lysate.

HRA-19, Colony 29 and Caco-2 cells were seeded onto tissue culture plastic or type I collagen-coated dishes (BioCoat) in serum-free medium. HRA-19 and Caco-2 cells showed a more flattened less cohesive morphology on type I collagen (unpublished observations), but Colony 29 cells displayed a dramatic scattering of cells not previously described for a colon cancer cell line (Figure 1A). Type I collagen also induced a downregulation of E-cadherin at cell–cell junctions in Colony 29, Caco-2 cells and HRA-19 cells (Figure 1B) although total E-cadherin protein was not reduced (Figure 1D). Interestingly, there were substantial differences in total E-cadherin between cell lines with a much lower level of E-cadherin in Colony 29 cells, the cell line showing the most marked morphological response to type I collagen (Figure 1D and A).

*β*-Catenin showed strong membrane expression at cell–cell contacts in Caco-2 cells on tissue culture plastic (Figure 1C), whereas Caco-2 cells on type I collagen showed a downregulation of cell–cell *β*-catenin and enhanced nuclear expression (Figure 1C). Colony 29 cells showed both cell–cell and nuclear *β*-catenin staining on plastic but on type I collagen; *β*-catenin was predominantly localised in the nucleus (Figure 1C). HRA-19 cells showed a strong cell–cell *β*-catenin expression on tissue culture plastic, with a small reduction seen in spread cells on type I collagen (Figure 1C), and nuclear *β*-catenin could only be discerned in isolated cells (white arrow; Figure 1C).

As type I collagen is the preferred ligand for *α*2*β*1 integrin (Kapyla *et al*, 2000), *α*2*β*1 integrin expression was also analysed. Although *α*2*β*1 integrin expression was similar on plastic or collagen, there were marked differences between cell lines (Figure 1D). It was notable that the cell line that had the most marked response to type I collagen, Colony 29, expressed much higher levels of *α*2 integrin (Figure 1D).

### Type I collagen inhibits lineage differentiation in human colorectal cancer cells

**Figure 2:**
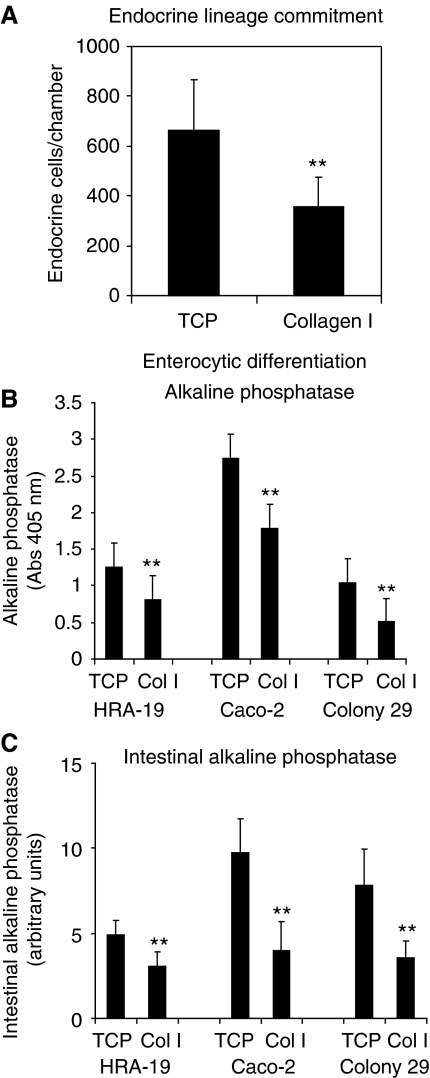
Collagen I inhibits lineage differentiation in human colorectal cancer cells. (**A**) Endocrine lineage commitment in multipotent HRA-19 cells when grown on tissue culture plastic (TCP) and type I collagen-coated plastic (collagen I). Equal numbers of cells were seeded at a 1 : 5 split ratio in eight-chamber slides, grown for 72 h in 10% FBS in DMEM and then transferred to serum-free medium (ITA) for 72 h. Monolayers were stained for chromogranin using immunocytochemistry and total endocrine cells counted in each chamber. Quadruplicate chambers were used for each condition, results shown are combined values from three independent experiments; mean±s.d. (*n*=3); ^**^P<0.001. (**B**) Alkaline phosphatase activity in HRA-19, Caco-2 and Colony 29 cells grown in plastic(TCP) or type I collagen (Col I)-coated 96-well plates for 72 h in serum-free medium (TS). Alkaline phosphatase absorbance values were normalised for cell number using the WST-1 cell-proliferation reagent (Roche). The experiment was performed in triplicate. Results shown are mean±s.d. (*n*=3) for three independent experiments; ^**^P<0.005. (**C**) Intestinal alkaline phosphatase expression in HRA-19, Caco-2 and Colony 29 cells grown in plastic (TCP) or type I collagen-coated 96-well plates for 48 h in serum-free medium (TS). Intestinal alkaline phosphatase was measured using a cell-based ELISA in triplicate with cell number normalised using Cyquant assay (Invitrogen). Results shown are mean±s.d. (*n*=3) for three independent experiments; ^**^P<0.005.

Type I collagen was shown to markedly inhibit endocrine lineage commitment in the multipotent human colorectal cancer line, HRA-19 (Figure 2A).

Type I collagen was also shown to inhibit expression of the enterocytic marker alkaline phosphatase (Matsumoto *et al*, 1990) in Caco-2, Colony 29 and HRA-19 cells using an enzymatic method for alkaline phosphatase assay (Figure 2B). Inhibition of enterocytic differentiation markers was confirmed by measuring intestinal alkaline phosphatase protein expression using a cell-based ELISA. Cell-based ELISA has been shown to be a sensitive assay for total (Versteeg *et al*, 2000) and cell surface (Hagi-Pavli *et al*, 2004) protein expression in intact cell monolayers. Intestinal alkaline phosphatase expression was shown to be markedly reduced in all cell lines when grown on type I collagen compared with plastic controls (Figure 2C).

### Type I collagen increases the expression of stem cell-associated markers CD133 and Bmi1

Recent studies have suggested that EMT is linked with the expression of a stem cell phenotype. To examine this possibility, cells undergoing EMT-like changes on type I collagen were analysed for expression of putative stem cell markers CD133 and Bmi1. An enhanced surface CD133 expression has been shown to characterise a sub-population of human colorectal cancer cells with tumour-initiating characteristics (O'Brien *et al*, 2007; Ricci-Vitiani *et al*, 2007). Bmi1 has been shown to be a marker of adult intestinal stem cells (Sangiorgi and Capecchi, 2008).

**Figure 3:**
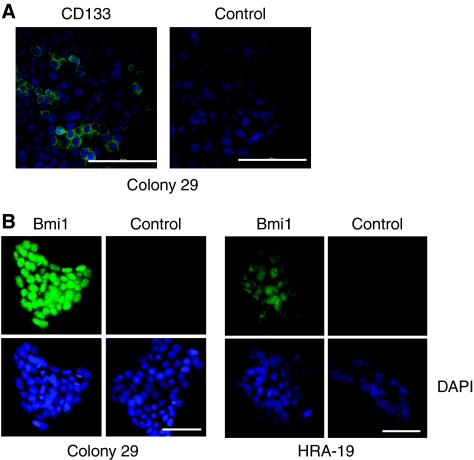
Colon cancer cell lines express stem cell-associated markers CD133 and Bmi1. (**A**) CD133 expression in Colony 29 grown in serum-free medium (TS) for 48 h. Cells were imaged using fluorescence microscopy. CD133 (green), nuclear counterstain DAPI (blue). Control=rabbit IgG control. Scale bar=100 *μ*m. (**B**) Bmi1 expression in Colony 29 cells and HRA-19 cells grown in serum-free medium (TS) for 72 h. Cells were imaged using fluorescence microscopy. Bmi1 (green) nuclear counterstain DAPI (blue). Control=rabbit IgG control. Scale bar=50 *μ*m.

CD133 and Bmi1 expression was first confirmed by immunocytochemistry. Colony 29 cells were shown to express CD133 in a sub-population of cells (Figure 3A) and Bmi1 was expressed by the majority of Colony 29 and HRA-19 cells (Figure 3B). Although staining appeared stronger in cells on type I collagen, the pattern of staining on plastic was indistinguishable from collagen cultures; therefore, just images from collagen cultures were used to illustrate the expression pattern. Western blots confirmed CD133 and Bmi1 expression in HRA-19, Colony 29 and Caco-2 cells (unpublished observations).

**Figure 4:**
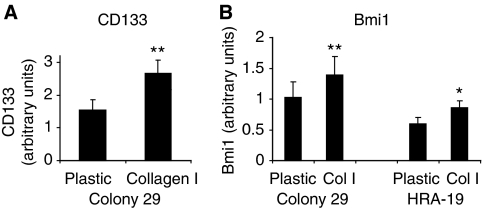
Type I collagen increases the expression of stem cell markers CD133 and Bmi1. (**A**) Cell surface expression of CD133 in Colony 29 cells grown in serum-free medium (TS) in plastic or collagen-coated 96-well plates for 72 h. Measurements were made in triplicate using a cell-based ELISA. Cells were fixed in 2% paraformaldehyde and not permeabilised to detect surface expression. Results presented are mean±s.d. of three independent experiments (*n*=3); ^**^P<0.005. (**B**) Bmi1 expression in Colony 29 and HRA-19 cells grown in serum-free medium (TS) in plastic or collagen-coated wells for 72 h. Measurements were made in triplicate using a cell-based ELISA. Results presented are mean±s.d. of three independent experiments (*n*=3); ^**^P<0.005; ^*^P<0.05.

CD133 and Bmi1 expression was measured quantitatively in a cell-based ELISA on tissue culture plastic or type I collagen. The surface expression of CD133 was determined in cells following fixation in paraformaldehyde without permeabilisation. Type I collagen was shown to promote the cell surface expression of CD133 in Colony 29 cells at 72 h (Figure 4A). Bmi1 expression was also increased in HRA-19 and Colony 29 cells when grown on type I collagen compared with tissue culture plastic (Figure 4B).

### Type I collagen increases clonogenicity in human colorectal carcinoma cells

**Figure 5:**
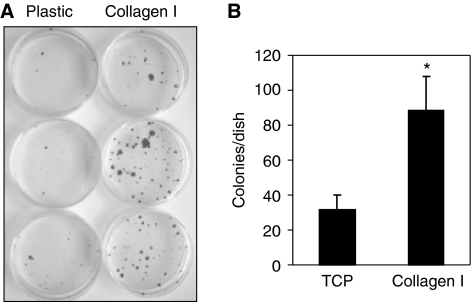
Collagen I increases clonogenicity in Caco-2 cells. Caco-2 cells (1 × 10^3^) were seeded in serum-free medium (TS) into plastic or type I collagen-coated (BioCoat) 6 cm dishes. Cells were fed twice weekly for 5 weeks, then fixed and stained with crystal violet. Colonies with greater than 10 cells were counted. A representative experiment shows increased colony formation on type I collagen-coated dishes (**A**) and the corresponding colony counts for this experiment (**B**); ^*^P<0.05. Similar experiments were repeated four times.

The clonogenicity of cells was compared on type I collagen-coated and plastic surfaces using the Caco-2 cell line, which was the only cell line from which a single-cell suspension could be generated. Caco-2 cells showed an enhanced ability for clonal growth when seeded on a type I collagen-coated surface in serum-free medium (Figure 5A and B). Colonies generated on type I collagen also appeared larger than those on plastic (Figure 5A).

### Type I collagen effects are mediated through *α*2*β*1 integrin

**Figure 6:**
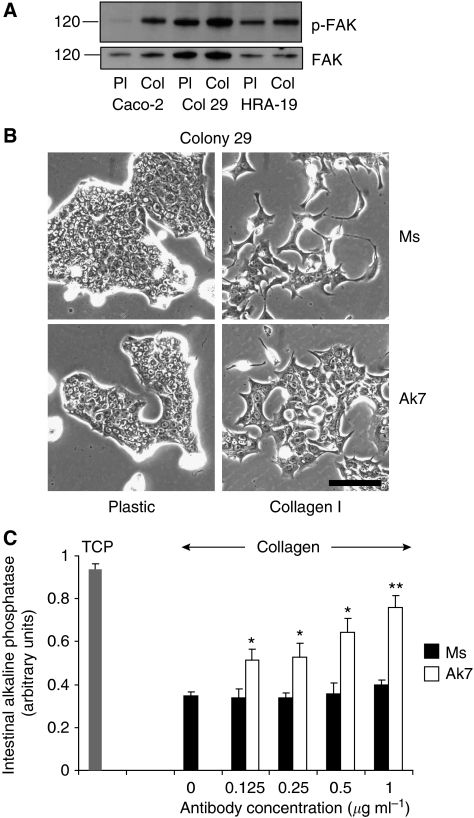
Collagen I effects are mediated through *α*2*β*1 integrin. (**A**) Western blot showing total FAK and p-FAK expression in 10 *μ*g cell lysate from Caco-2, Col 29 and HRA-19 cells after 2 h on tissue culture plastic (Pl) or type I collagen-coated plastic (Col). (**B**) Phase contrast micrographs of Colony 29 cells grown in serum-free medium on tissue culture plastic (plastic) or type I collagen-coated plastic dishes for 48 h at 37 °C in the presence of either Ms IgG control (Ms) or anti-*α*2 integrin (Ak7) at 300 ng ml^−1^. Experiment was performed twice. Scale bar=100 *μ*m. (**C**) Cell-based ELISA for intestinal alkaline phosphatase activity in Colony 29 cells grown on TCP or type I collagen-coated wells (collagen) in the presence of varying concentrations of Ms IgG (Ms) or anti-*α*2 integrin (Ak7). Values were normalised for cell number using WST-1 reagent. Triplicate wells were used for each condition. Results shown are mean±s.d. ^**^P<0.005; ^*^P<0.05. Other experiments with individual concentrations of antibody were performed at least three times with similar results.

The interaction of colon cancer cells with collagen through *α*2*β*1 integrin leads to the phosphorylation of focal adhesion kinase (FAK) at Tyr^397^ (Sawhney *et al*, 2006). FAK^397^ was found to be more highly phosphorylated in all cell lines on type I collagen (Figure 6A), suggesting that integrin signalling was involved in the cellular responses to collagen.

To directly assess the role of *α*2*β*1 integrin in EMT-like phenotypic changes, Colony 29 cells were incubated with type I collagen in the presence of an *α*2 integrin function-blocking antibody, AK7, or a Ms IgG isotype control at the same concentration. Type I collagen induced a cell scattering in control cells, whereas Ak7 was shown to partially block the acquisition of this distinctive migratory phenotype in Colony 29 cells (Figure 6B). Enterocytic differentiation was also measured in cells on plastic and on type I collagen in the presence of *α*2 integrin antibody, Ak7, or Ms IgG control. Cells grown in control antibody showed the previously described reduction in differentiation on type I collagen, whereas the addition of Ak7 partially abrogated this effect (Figure 6C). These results suggest that the type I collagen-induced effects on the human colorectal cancer cell phenotype are mediated, at least in part, by signalling through *α*2*β*1 integrin.

## Discussion

Transient changes in cancer cell phenotype are observed at the invasive edge of human colorectal cancers, suggesting that the microenvironment regulates tumour progression (Brabletz *et al*, 2001). Type I collagen is abundantly expressed at this invasive front and preliminary studies have suggested that it is responsible for the loss of differentiation and EMT-like changes seen in this area (Brabletz *et al*, 2004). This study confirms and extends these findings, showing that human colorectal carcinoma cell lines undergo the initial steps of EMT when plated onto type I collagen. These EMT-like changes include a spectrum of morphological changes between cell lines ranging from a more flattened and less cohesive morphology to the dramatic scattered morphology seen in Colony 29. Further evidence of EMT-like changes on type I collagen was provided by the downregulation of E-cadherin at cell–cell junctions as shown previously in pancreatic carcinoma cells (Grzesiak *et al*, 2005; Koenig *et al*, 2006). In pancreatic carcinoma cells, some studies show an additional decrease in E-cadherin protein on type I collagen (Koenig *et al*, 2006). In this study, the downregulation of E-cadherin at cell–cell junctions was not accompanied by a decrease in E-cadherin protein expression in agreement with other studies on pancreatic carcinoma cells (Shintani *et al*, 2006). However, constitutive levels of E-cadherin were shown to be much lower in Colony 29 cells than in Caco-2 or HRA-19, raising the possibility that this inherent characteristic rendered the cells more responsive to type I collagen. Some evidence to support this notion comes from work on the HCA-7 cell line, the parent line of Colony 29 cells, which also shows a low level of E-cadherin protein expression (Chang *et al*, 2006). In HCA-7 cells, E-cadherin is downregulated by constitutive expression of COX-2 and RhoA, which are associated with the disruption of adherens junctions in these cells (Chang *et al*, 2006). COX-2-dependent pathways upregulate ZEB1 and Snail, transcriptional suppressors of E-cadherin in lung cancer cells (Dohadwala *et al*, 2006). ZEB1 represses differentiation and cell–cell adhesion in human colorectal carcinoma cells (Aigner *et al*, 2007). In addition, a stable COX-2 expression in breast epithelial cells enhances EMT (Neil *et al*, 2008). Therefore, it seems likely that the elevated levels of COX-2 in Colony 29 cells (Chinery *et al*, 1999) are involved in enhancing the response of these cells to type I collagen.

*β*-Catenin has dual functions in cell–cell adhesion and transcription, with an imbalance between these two functions leading to cancer formation (Harris and Peifer, 2005). Nuclear *β*-catenin, an indicator of active Wnt signalling, acts as a transcriptional activator in concert with TCF proteins and is both important in normal stem cell renewal and the main oncoprotein in human colorectal cancer (Peifer, 1997). The exposure of Colony 29 and Caco-2 cells to type I collagen resulted in a loss of *β*-catenin at cell–cell contacts and an increase in nuclear *β*-catenin. Nuclear *β*-catenin is observed in the invasive cells of human colorectal cancer (Brabletz *et al*, 2001), suggesting that the exposure of cancer cells to type I collagen at the invasive edge enhances nuclear *β*-catenin expression. Nuclear *β*-catenin has been shown to impose an undifferentiated progenitor phenotype on colorectal cancer cells (van de Wetering *et al*, 2002); therefore, the differentiation and expression of stem cell characteristics were compared between cells growing on plastic and type I collagen.

Type I collagen has previously been shown to reduce cdx-2 expression in colon cancer cells (Brabletz *et al*, 2004). This study confirms and extends this earlier study by showing that type I collagen inhibits enterocytic differentiation in all cell lines tested. The study was extended to compare cell fate decisions by multipotent colorectal cancer cells (HRA-19) when grown on tissue culture plastic and type I collagen. Endocrine lineage commitment was inhibited by the growth of cells on type I collagen, suggesting that type I collagen promotes self-renewal, maintaining colorectal carcinoma cells in an undifferentiated progenitor state.

This study shows that type I collagen induces EMT-like changes and a loss of differentiation in human colorectal carcinoma cells. Interestingly, recent studies have linked EMT with the acquisition of stem cell properties in mammary epithelial cells (Mani *et al*, 2008). Therefore, stem cell marker expression was investigated to determine whether the EMT-like changes observed on collagen were similarly associated with a stem cell-like phenotype. Two stem cell markers, CD133 and Bmi1, were chosen and Colony 29 cells investigated in greater detail as they displayed the greatest EMT-like response to type I collagen. Cell surface CD133 has been shown to be a stem cell marker in normal and neoplastic cells (Mizrak *et al*, 2008), including human colorectal cancer stem cells (O'Brien *et al*, 2007; Ricci-Vitiani *et al*, 2007).Cell surface expression of CD133 was increased on Colony 29 cells grown on type I collagen, suggesting that type I collagen maintains the stem cell phenotype. Further evidence for this was sought using another stem cell marker, Bmi1, which has a role in self-renewal in a variety of stem cells and has recently been shown to be an intestinal stem cell marker (Sangiorgi and Capecchi, 2008) . Colony 29 cells also showed increased Bmi1 expression when grown on type I collagen, further supporting the idea that cells were expressing a more stem cell-like phenotype. Increased Bmi1 expression was also observed in HRA-19 cells on type I collagen. Bmi1 is involved in self-renewal in haematopoietic and neuronal cells (Lessard and Sauvageau, 2003; Molofsky *et al*, 2003). In addition, Bmi1 expression is dysregulated in preneoplastic colorectal epithelium and overexpression correlates with malignant progression (Tateishi *et al*, 2006), supporting a role for Bmi1 in human colorectal cancer progression. Previous studies have shown that HRA-19 cells, which have a low inherent tumourigenicity, acquired a 100% take rate in xenografts when embedded in type I collagen gel (Del-Buono *et al*, 1991), suggesting a functional significance to colon cancer cell–collagen interactions. To examine whether an increased expression of stem cell markers was accompanied by any functional attributes of stem cells, clonogenicity on type I collagen was determined. Clonogenicity is associated with the stem cell phenotype and was enhanced in Caco-2 cells when grown on type I collagen.

We have recently shown that *α*2*β*1 integrin regulates cell fate decisions in the multipotent colorectal cancer cell line, HRA-19 (Kirkland and Ying, 2008).This study provides evidence that *α*2*β*1 integrin is involved in type I collagen-induced EMT-like changes and loss of differentiation. However, Ak7, an *α*2 function-blocking antibody, was only able to partially abrogate type I collagen-induced effects on colon cancer cell phenotype, suggesting the possible involvement of other collagen receptors. For example, it has been shown recently that *α*2*β*1 integrin cooperates with Discoidin domain receptor I, a tyrosine kinase collagen receptor, to increase N-cadherin expression in pancreatic cancer cells (Shintani *et al*, 2008).

In this study, the interaction between colon cancer cells and type I collagen results in EMT-like changes, a loss of differentiation and an increased expression of stem cell markers. These phenotypic changes are all characteristics of cancer cells at the invasive edge of human colorectal cancers. The link between EMT-like changes and expression of a stem cell phenotype supports the concept of a migrating cancer stem cell being involved in tumour progression and dissemination (Brabletz *et al*, 2005). Overall, the results indicate that type I collagen promotes the expression of a more malignant, stem cell-like phenotype in human colorectal cancer cells.
